# Endoscopic retrograde cholangiopancreatography in adult patients with biliary atresia: PROCESS-compliant case series

**DOI:** 10.1097/MD.0000000000010603

**Published:** 2018-05-04

**Authors:** Jong Jin Hyun, Shayan S. Irani, Richard A. Kozarek

**Affiliations:** a Digestive Disease Institute, Virginia Mason Medical Center, Seattle, WA; b Department of Internal Medicine, Korea University College of Medicine, Seoul, Korea.

**Keywords:** ascending cholangitis, biliary atresia, endoscopic retrograde cholangiopancreatography, Kasai portoenterostomy

## Abstract

**Introduction:**

Biliary atresia is a progressive inflammatory disease of the bile duct that eventually results in biliary cirrhosis. It is a rare neonatal disease that mandates treatment within the first 2 years of life in order for the infant to survive. Patients usually undergo palliative Kasai portoenterostomy. Even when Kasai portoenterostomy has been performed in a timely manner, progression is still inevitable. In fact, the majority of patients require curative liver transplantation at a later stage before reaching adulthood.

**Methods:**

Two jaundiced biliary atresia patients who have lived well beyond 20 years with their native liver after undergoing Kasai portoenterostomy and underwent endoscopic retrograde cholangiopancreatography (ERCP) were identified. The data on patients’ clinical information, procedures performed, and outcomes were retrospectively collected by chart review.

**Results:**

Presence of a long Roux limb and acute angulation from external adhesions along with ductal anomaly from disease itself rendered ERCP challenging, and intraoperative ERCP had to be performed in 1 patient. As enteroscopes had to be used, availability of accessory devices was limited.

**Conclusion:**

Management of adult biliary atresia patients with biliary obstruction with ERCP is feasible, at times, through multidisciplinary means.

## 1 Introduction

Biliary atresia is a rare neonatal disease of both intrahepatic and extrahepatic bile ducts, which to a varying extent become obliterated by an inflammatory destructive process. The latter causes severe cholestasis, eventually leading to biliary cirrhosis. If left untreated, biliary atresia results in death within the first 2 years of life.^[1]^ Therefore, the presence of acholic stool, dark urine, hepatomegaly, and neonatal jaundice persisting beyond 14 days should arouse suspicion and pertinent investigation should be pursued.^[2]^ When further biochemical, radiologic, and/or pathologic evaluation suggest the presence of biliary atresia, exploratory laparotomy is performed with the intention to surgically correct this condition. Operative cholangiography is obtained when doubt exists about the definite diagnosis and also to document the site of obstruction. Once the diagnosis is made, the initial treatment of choice is the Kasai portoenterostomy, which was developed by a Japanese surgeon Morio Kasai in 1959.^[3]^ Kasai portoenterostomy aims to re-establish bile flow by resecting the damaged extrahepatic bile ducts up to the liver capsule and anastomosing the exposed porta hepatis to a jejunal Roux-en-Y limb. As Kasai portoenterostomy is a palliative procedure, it cannot halt the liver from progressing to biliary cirrhosis. Although modifications have been made to this operative procedure, progression is the rule and patients eventually require liver transplantation. Indeed, after Kasai portoenterostomy, nearly half of the patients require liver transplantation within 2 years, approximately one-third of patients (range 13% to 60%) live to be 10 years old without liver transplantation, and only about one-fourth of patients (range 15% to 48%) are alive with their native liver at 20 years of age.^[4,5]^ The authors report 2 cases of biliary atresia patients who survived well past 20 years of age with their native liver but presented with biliary obstructive symptoms.

## 2 Methods

We identified 2 adult patients with biliary atresia who required endoscopic retrograde cholangiopancreatography (ERCP) to maintain biliary patency. The data were obtained by retrospectively reviewing the electronic medical records. This study was reviewed by the institutional review board of Virginia Mason Medical Center, but ethical approval was waived, as it involved data acquisition by chart review without any patient contact.

### 2.1 Case 1

A 30-year-old male patient with a history of having undergone Kasai procedure at 4 weeks of age due to congenital biliary atresia was admitted with acute abdominal pain and nausea. At admission, his vital signs were stable (116/71 mm Hg – 36.5°C – pulse rate: 76/min – respiratory rate: 16/min). Physical examination was remarkable only for minimal right upper quadrant tenderness. His initial laboratory results were as follows: white blood cell count, 4300/μL (neutrophil 70.3%); hemoglobin, 13.6 g/dL; platelet, 139,000/μL; serum protein, 5.4 g/dL (6.4–8.3 g/dL); serum albumin, 3.1 g/dL (3.5–5.2 g/dL); total bilirubin, 5.2 mg/dL (0.2–1.2 mg/dL); direct bilirubin, 3.4 mg/dL (0.1–0.5 mg/dL); aspartate transaminase (AST), 18 IU/L (10–40 IU/L); alanine transaminase (ALT), 115 IU/L (10–55 IU/L); alkaline phosphatase (ALP), 26 IU/L (40–150 IU/L). For further evaluation and management, ERCP using a single balloon enteroscope (SBE) was performed following nondiagnostic computed tomography (CT) scan and magnetic resonance cholangiopancreatography (MRCP).

### 2.2 Case 2

A 24-year-old female with a history of having undergone Kasai procedure at 8 weeks of age for biliary atresia was admitted because of ongoing right upper quadrant pain. The patient had intermittent right upper quadrant abdominal pain, pruritus with intermittent rigors, and low-grade fevers for the past 6 months. At admission, her vital signs were stable (128/77 mm Hg – 36.8°C – pulse rate: 88/min – respiratory rate: 18/min) and physical examination was unremarkable. Laboratory results were as follows: white blood cell count, 7800/μL(neutrophil 53.7%); hemoglobin, 13.3 g/dL; platelet, 219,000/μL; total bilirubin, 0.5 mg/dL (0.2–1.2 mg/dL); AST, 44 IU/L (10–40 IU/L); ALT, 76 IU/L (10–55 IU/L); ALP, 216 IU/L (40 – 150 IU/L). Abdominal ultrasound and MRCP performed at outside hospital showed no definite obstructive lesion. Hepatobiliary scan also failed to demonstrate biliary obstruction. Therefore, the patient underwent ERCP using a double-balloon enteroscope (DBE).

## 3 Results

### 3.1 Case 1

**Figure 1:**
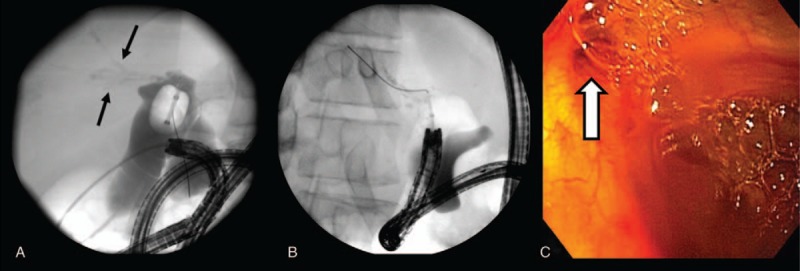
Separate intrahepatic ducts are noted around the anastomosis site (A, black arrows). The ducts were catheter dilated using a 6 to 8 mm CRE balloon on a 6-French catheter (B). Large amount of sludge and air bubbles are seen coming out from the biliary orifice (white arrow) after catheter dilatation (C).

There were no visible esophageal varices or portal gastropathy upon insertion of the scope. On ERCP, at least 3 separate stenotic hepaticojejunostomies consistent with biliary atresia and anastomoses of small intrahepatic ducts to the Roux limb were noted (Fig. 1A). These were catheter dilated using a 6 to 8 mm continuous radial expansion (CRE) balloon on a 6-French catheter, but the ducts were so small that it was felt unsafe to inflate the balloon (Fig. 1B). The intrahepatic ducts that were seen were sclerotic and filled with debris. After dilation, copious debris and air bubbles as well as sludge was noted to egress into the jejunum (Fig. 1C). The patient's condition improved and laboratory findings normalized after the procedure. He was followed-up for 2 years after endoscopic therapy and during that period, had several episodes of transient cholangitis but responded well to antibiotics.

### 3.2 Case 2

**Figure 2:**
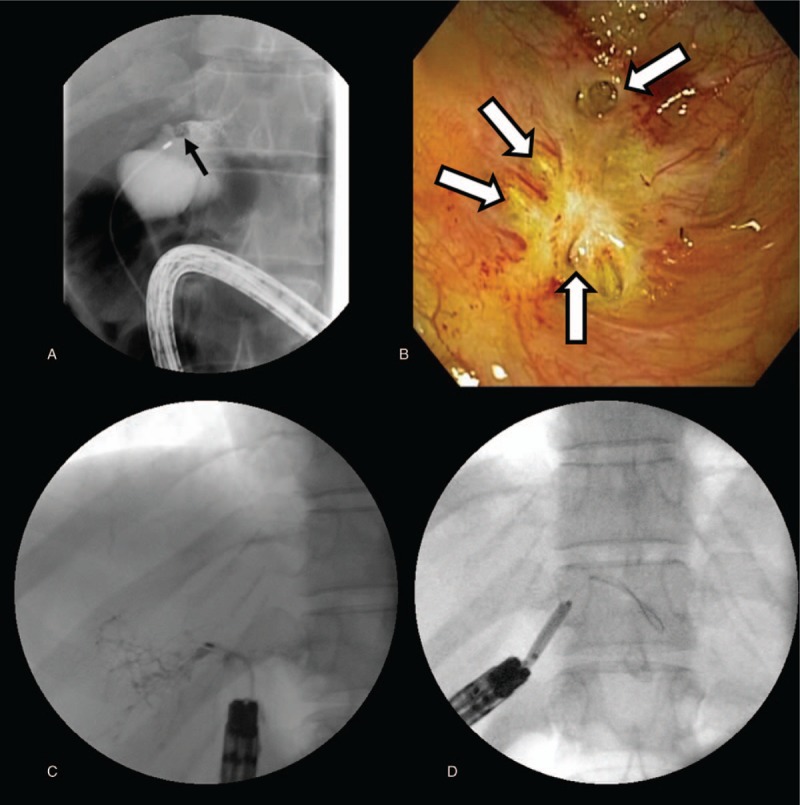
A 5–6 mm sized square filling defect is noted near the orifice of left ducts (black arrow), but removal of the stone was not feasible (A). The scope could approach the anastomosis site during intraoperative ERCP and multiple separate openings with fibrotic tissue are demonstrated (B, white arrows). Several of the structured ducts were dilated with catheter (C) and balloon (D).

The scope could be passed within 30 cm of the anastomosis but could not be advanced further. A guidewire was placed followed by an ERCP catheter and contrast injection demonstrated multiple, isolated ducts consistent with a Kasai procedure. A 5 to 6 mm square stone was observed obstructing one of the dilated left ducts (Fig. 2A). However, stone removal could not be performed because additional attempts to pass the scope further proved unsuccessful. Three months later, she underwent intraoperative ERCP facilitated by laparoscopic transjejunal entry site using a SBE. The scope could be passed to the Roux limb closure site and multiple tiny ducts anastomosed to the Roux limb at the level of the hilum were documented (Fig. 2B). Several of the ductal systems were dilated with a 5-French catheter with difficulty (Fig. 2C). Dilating balloons of 4 mm were also used to dilate the ducts and resulted in passage of significant debris (Fig. 2D), but complete dilation of the intrahepatic ducts proved impossible, as passing a dilating balloon into multiple stenoses was not feasible. Nevertheless, the patient dramatically improved after the procedure. Although the patient has had intermittent pruritus and liver enzyme elevation after endoscopic therapy, she has responded well to ursodeoxycholic acid is currently being followed-up at outpatient clinic 6 years after endoscopic manipulation.

## 4 Discussion

The outcome of Kasai portoenterostomy seems to have been favorable for the 2 patients in the current report, as they managed to survive with their native liver for more than 20 years. Several factors that influence the outcome after Kasai procedure include the age at the time of Kasai portoenterostomy, biliary atresia type, and the experience of the surgical center.^[6,7]^ Although both patients in the present report had type III biliary atresia, which is a less favorable factor compared with type I or II biliary atresia, having performed Kasai portoenterostomy at a relatively young age seems to have acted in favor of prolonging survival with their native liver. There is also a possibility that ascending cholangitis, which occurs in 40% to 93% of patients during the first year after the operation, had been well controlled in the past.^[8]^ Past history of not having been frequently treated or hospitalized for cholangitis up to the point of undergoing endoscopic examination may have been another factor that contributed to good prognosis, as the number of cholangitis episodes has been reported to be inversely related to the success of Kasai portoenterostomy.^[9]^

Post-Kasai cholangitis is initially managed by intravenous antibiotics, but when there is a lack of response to medical therapy and/or imaging studies indicate the possibility of mechanical obstruction, biliary drainage should be considered. Biliary drainage has traditionally been carried out via percutaneous route or by surgical revision, but advances in deep enteroscopy have enabled endoscopic therapy, which has the advantage of not needing external drainage and being less invasive. Nevertheless, ERCP using either SBE or DBE is not always feasible due to the presence of a long Roux limb and acute angulation from external adhesions. The success rate of reaching the papilla and/or biliary anastomosis site using SBE or DBE in patients with Roux-en-Y enteroenteric anastomosis has been reported to range from 71% to 85.3%.^[10–12]^ In fact, one of the patients (case 2) in the present report had to undergo intraoperative ERCP, as the scope failed to reach the anastomosis site. Once the anastomosis site was reached, cannulation posed another problem, as the bile ducts have a small caliber, are markedly fibrotic, and usually separated in post-Kasai biliary atresia patients. Therefore, not all ducts can be located or cannulated. For these reasons, the success rate of obtaining a cholangiogram during ERCP in patients with Roux-en-Y anastomosis has been reported to be lowest in those who had undergone childhood surgery (38%), especially Kasai portoenterostomy (20%).^[10]^ Even after achieving successful cannulation, endoscopists are limited in the choice of accessories, as the length of the devices commonly used during ERCP fall short in this setting. Therefore, a CRE balloon dilation catheter (Boston Scientific, Natick, MA) was used to dilate the strictures and remove the sludge/stones in the present cases. Due to the small duct diameter, however, the orifice was dilated without inflating the balloon for fear for inducing ductal damage in case 1, although balloon dilatation could safely be performed in case 2.

After relieving the mechanical obstruction, efforts should be put into preventing or lowering the future episodes of recurrent cholangitis. The mechanism of ascending cholangitis is not entirely understood, but intestinal bacterial stasis and incomplete biliary drainage are considered to play an important role.^[13]^ Therefore, some centers use rotating antibiotics for intestinal decontamination and other centers advocate the use of ursodeoxycholic acid (20 mg/kg/day) alone or in conjunction with corticosteroids to enhance bile flow and to inhibit the inflammatory response. Although controlled studies looking into the efficacy of these prophylactic measures are either lacking or shown to be ineffective,^[14,15]^ they are commonly employed and favored by clinicians because no other interventions are currently available. With no efficacious alternatives at hand, prophylactic measures were also given to our patients, and several episodes of cholangitis after endoscopic therapy could be well controlled by antibiotics in case 1, and fluctuating symptoms, mainly pruritus, and liver function enzyme elevation could be managed fairly well with ursodeoxycholic acid in case 2.

As biliary atresia is a progressive disease, development of portal fibrosis, cirrhosis, and portal hypertension along with its complications are inevitable.^[1]^ Although the time to liver transplantation can be delayed by effectively controlling cholangitis, patients will eventually require liver transplantation when there is persistent liver function deterioration due to progression of biliary cirrhosis or develop intractable complications of portal hypertension unresponsive to other measures.^[16]^ The liver functions in patient number 2 improved but did not normalize with the administration of ursodeoxycholic acid, albeit symptoms were ameliorated. Both patients are currently being closely monitored to detect signs of hepatic decompensation so that liver transplantation can be performed in a timely manner, if necessary. The prognosis after liver transplantation is very good with dramatic improvement in survival rate, which now reaches over 90% in industrialized countries.^[16]^

## 5 Conclusion

ERCP is feasible in adult patients with biliary atresia post-Kasai, at times through a multidisciplinary approach. As biliary atresia is a progressive disease and deterioration of the liver function generally occurs with time, recurrent cholangitis should be adequately controlled to delay the time from palliative Kasai portoenterostomy to curative liver transplantation. Endoscopic therapy appears to be a reasonable therapeutic modality, as it carries less morbidity than a percutaneous approach in the setting of nondilated biliary ducts and obviates external drainage.

## Author contributions

**Conceptualization:** Richard A. Kozarek.

**Data curation:** Jong Jin Hyun, Shayan S. Irani, Richard A. Kozarek.

**Methodology:** Richard A. Kozarek.

**Writing – original draft:** Jong Jin Hyun.

**Writing – review & editing:** Shayan S. Irani, Richard A. Kozarek.
